# Editorial: Exploring microbial interactions in pancreatic ductal adenocarcinoma microenvironment

**DOI:** 10.3389/fonc.2025.1725126

**Published:** 2025-11-04

**Authors:** Serena Veschi, Pierluigi Di Sebastiano, Gabriella Mincione

**Affiliations:** ^1^ Department of Pharmacy, University “G. d’ Annunzio” of Chieti-Pescara, Chieti, Italy; ^2^ Department of Innovative Technologies in Medicine & Dentistry, University “G. d’Annunzio” of Chieti-Pescara, Chieti, Italy

**Keywords:** pancreatic ductal adenocarcinoma (PDAC), microbiota, tumor microenvironment, immunology, metabolites, precision oncology

Pancreatic ductal adenocarcinoma (PDAC) remains among the most lethal malignancies, with limited therapeutic options and only modest survival improvements despite advances in systemic chemotherapy and surgical techniques (1). In recent years, the tumor microenvironment (TME) has emerged as a key determinant of PDAC progression and therapeutic resistance (2). Among the multiple components shaping the TME, the microbiota—both local and systemic—has gained increasing attention for its potential to modulate immune responses, drug metabolism, and tumor biology. This Research Topic brings together four complementary contributions, encompassing clinical, preclinical, and review studies, that collectively highlight the multifaceted and sometimes ambivalent role of the microbiota in PDAC.

Microbiome disruption, or dysbiosis, has been implicated in a wide range of pathological conditions, underscoring the importance of elucidating its characteristics and functions in health and disease (3). The contribution of the human microbiota to pancreatic carcinogenesis has long been hypothesized, although the underlying mechanisms remain largely undefined (4). The microbiota plays a critical role in modulating immune responses, nutrient metabolism, and epithelial barrier integrity. Notably, the skin harbors a complex microbial community known as the skin microbiota (5). Within this context, Davis et al. conducted a pilot study analyzing the skin microbiome of patients with PDAC in comparison with individuals affected by other cancer types and healthy controls. Using swab-based sampling and next-generation sequencing, the authors demonstrated significantly greater skin microbial diversity in cancer patients. Machine learning models trained on microbial abundance profiles achieved high accuracy (F1 score ≈0.94) in distinguishing cancer from control cohorts. Specific taxa, including *Veillonella atypica*, *Streptococcus suis*, and *Klebsiella pneumoniae*, were enriched in cancer patients, whereas *Cutibacterium acnes* SK182 predominated among healthy individuals. These findings suggest that skin microbiome profiling may serve as a non-invasive biomarker platform, reflecting systemic host–microbiota alterations associated with PDAC malignancy.

Recent evidence indicates that microbial metabolites can influence treatment outcomes in PDAC. In metastatic disease, the gut-derived metabolite indole 3-acetate (3-IAA) was reported to enhance chemotherapy response by modulating oxidative stress and neutrophil activity (6). In this Research Topic, Braadland et al. investigated whether circulating 3-IAA levels could predict therapeutic response in patients with borderline resectable or locally advanced PDAC. In a prospective cohort of 124 patients, baseline serum 3-IAA levels showed no significant association with overall survival, even after adjustment for clinical covariates. These results contrast with those reported by Tintelnot et al. (6), suggesting that the prognostic relevance of 3-IAA may be stage-specific or context-dependent. The study underscores the heterogeneity of microbiota–host interactions across disease settings and highlights the need for biomarker validation across independent cohorts. Although preliminary, these findings support further exploration of microbiome composition and metabolite profiles across tumor stages and anatomical niches. Such efforts could foster the development of microbiome-based, non-invasive biomarkers for the diagnosis and monitoring of PDAC and other malignancies.

PDAC progression is also characterized by profound immune tolerance, involving alterations in the number, phenotype, and function of immune cell subsets, particularly myeloid populations that contribute to the establishment of an immunosuppressive microenvironment (7). In a preclinical study, Daniluk et al. employed genetically engineered mice carrying oncogenic *KRAS* mutations—a hallmark of PDAC. When combined with inflammatory stimuli such as cerulein, this genetic predisposition accelerated the transition from chronic pancreatitis to pancreatic intraepithelial neoplasia (PanIN) and ultimately to invasive carcinoma. Remarkably, fecal microbiota transplantation from tumor-bearing mice further promoted tumor development in susceptible hosts, providing mechanistic evidence for a causal role of the microbiota in disease progression. Immune profiling revealed marked alterations in dendritic cells, myeloid populations, and T-cell subsets, consistent with a shift from immune surveillance to tolerance. These findings mirror human data describing PDAC as an “immune-cold” tumor, dominated by regulatory T cells, M2 macrophages, and myeloid-derived suppressor cells. Overall, this study establishes a functional link between microbiota, inflammation, and immune dysfunction, offering valuable insights for microbiota-targeted immunotherapeutic strategies.

Finally, Sindaco et al. provide a comprehensive review summarizing current knowledge on the relationship between the microbiota and PDAC pathogenesis, progression, and therapy. The authors describe how intra-tumoral, gastrointestinal, and oral microbial communities influence carcinogenesis from early precursor lesions (IPMNs and PanINs) to advanced disease. Dysbiosis contributes to oxidative stress, immune evasion, and epithelial–mesenchymal transition, while specific bacterial, fungal, and viral taxa can exert either tumor-promoting or tumor-protective effects. Distinct microbial signatures have also been identified between long-term and short-term survivors, suggesting potential prognostic utility—genera such as *Saccharopolyspora*, *Pseudoxanthomonas*, and *Bacillus* being associated with improved outcomes. The review highlights translational opportunities, including the integration of microbiota-derived markers with CA19–9 for early detection and the use of fecal or salivary microbial profiling as non-invasive diagnostic tools. Moreover, therapeutic modulation through antibiotics, probiotics, fecal microbiota transplantation, or microbial vectors may enhance the efficacy of chemotherapy and immunotherapy. Nonetheless, the authors emphasize the “double-edged” nature of microbiota manipulation: while certain microbes sensitize tumors to therapy, others may promote progression, calling for cautious and context-specific clinical applications.

Collectively, these studies underscore the intricate interplay between the microbiota and PDAC, spanning disease pathogenesis, immune modulation, and therapeutic response. A deeper understanding of these relationships may pave the way toward precision-medicine strategies that integrate microbial, immune, and metabolic profiling to improve clinical outcomes in pancreatic cancer.

## Lessons across studies: from observation to translation

Taken together, the four contributions illustrate the multifaceted ways in which microbiota interact with PDAC:

- At the systemic level, as suggested by skin microbiome alterations, potentially useful for early, non-invasive detection.
- At the metabolic level, where microbial metabolites like 3-IAA may shape therapeutic efficacy.
- At the mechanistic level, where microbial communities directly influence immune dynamics in genetically susceptible hosts.
- At the integrative level, as summarized by Sindaco et al., who critically reviewed recent findings and emphasized the ambivalent—”double-edged”—nature of microbiota, highlighting both tumor-promoting and tumor-protective roles, as well as their potential as diagnostic, prognostic, and therapeutic tools.

These layers of evidence converge toward a common message: the microbiota is not a passive bystander but an active participant in PDAC biology.

## Future directions and clinical implications

Despite promising data, several challenges remain before microbiota-based strategies can be translated into clinical practice. Key open questions include:

1. Biomarker development – Can microbial signatures be validated in large, independent cohorts as reliable diagnostic or prognostic tools?
2. Therapeutic modulation – Which strategies (antibiotics, probiotics, dietary interventions, fecal microbiota transplantation) are both safe and effective in modulating PDAC progression or therapy response?
3. Mechanistic clarity – What are the molecular mediators of microbiota-host crosstalk in the pancreatic TME, and how can they be targeted?
4. Integration with precision oncology – How can microbiome profiling be incorporated into multi-omic frameworks that already include genomics, transcriptomics, and metabolomics?

Addressing these questions will require multidisciplinary collaborations, standardized protocols, and careful consideration of patient safety.

## Conclusion

PDAC continues to pose formidable clinical and biological challenges. The works presented in this Research Topic highlight how microbiota research is reshaping our understanding of PDAC pathogenesis, immune evasion, and therapy resistance. By bridging clinical observations, preclinical mechanistic models, and critical reviews, these studies provide a roadmap for future investigations.

Ultimately, clarifying the relationship between the PDAC–microbiota axis may open avenues for earlier diagnosis, more precise patient stratification, and innovative therapeutic strategies. As our knowledge deepens, what is now one of the most intractable malignancies may gradually become more amenable to microbiota-informed precision oncology.
